# Design Features of a Titanium Mesh for Guided Bone Regeneration and In Vivo Testing in Vitamin D3 Deficiency Condition

**DOI:** 10.3390/biomimetics11020091

**Published:** 2026-01-28

**Authors:** Ekaterina Diachkova, Aglaya Kazumova, Andrei Shamanaev, Liubov Shcherbinina, Alexandr Gulyaev, Yuriy Vasil’ev, Pavel Petruk, Anzhela Brago, Yulianna Enina, Valerii Chilikov, Hadi Darawsheh, Ekaterina Makeeva, Svetlana Tarasenko

**Affiliations:** 1 Department of Oral Surgery, Borovskiy Institute of Dentistry, I.M. Sechenov First Moscow State Medical University (Sechenov University), 8/2 Trubetskaya St., Moscow 119991, Russia; aglaya.kazumowa@yandex.ru (A.K.); tarasenko_s_v@staff.sechenov.ru (S.T.); 2 Department of Operative Surgery and Topographic Anatomy, Sklifosovskiy Institute of Clinical Medicine, I.M. Sechenov First Moscow State Medical University (Sechenov University), 8/2 Trubetskaya St., Moscow 119991, Russia; vasilev_yu_l@staff.sechenov.ru (Y.V.); enina_yu_i@staff.sechenov.ru (Y.E.); chilikov_v_v@staff.sechenov.ru (V.C.); hadi.darawsheh@gmail.com (H.D.); 3 Medical Faculty, Sklifosovskiy Institute of Clinical Medicine, I.M. Sechenov First Moscow State Medical University (Sechenov University), Trubetskaya St., 8, Building 2, Moscow 119991, Russia; shamanaev143@mail.ru (A.S.); lubashcherbinina26@gmail.com (L.S.); 4 Medical Faculty, Russian University of Medicine, Dolgorukovskaya Street 4, Moscow 127006, Russia; guliyaev12345@yandex.ru; 5 Maxillofacial Surgery Department, Borovskiy Institute of Dentistry, I.M. Sechenov First Moscow State Medical University (Sechenov University), 8/2 Trubetskaya St., Moscow 119991, Russia; petruk_p_s@staff.sechenov.ru; 6 Department of Propedeutics of Dental Diseases, Medical Institute, Peoples’ Friendship University of Russia Named After Patrice Lumumba, Moscow 117198, Russia; anzhela_bogdan@mail.ru; 7 Anatomy Department, Russian University of Medicine, Dolgorukovskaya Street 4, Moscow 127006, Russia; makeevi@inbox.ru

**Keywords:** guided bone regeneration, individualized titanium mesh, alveolar process atrophy, 3D printing, selective laser sintering, bone grafting, dental implantation

## Abstract

Prolonged tooth loss causes alveolar ridge atrophy, complicating implantation, especially in patients with impaired mineral metabolism. This study aimed to develop a personalized titanium mesh for guided bone regeneration and qualitatively evaluate its local tissue response in a vitamin D3-deficient rabbit model. A titanium mesh design has been developed in the form of a plate-shaped profile frame of a truncated pyramid with a solid upper base and perforated side faces. For testing in a rabbit model with vitamin D3 deficiency, a bone defect was created and repaired in the mandible using hydroxyapatite, an individual titanium mesh and a collagen membrane. Histological analysis was performed in the Laboratory of Digital Microscopic Analysis. The optimized geometry and parameters of the mesh openings contributed to effective vascularization and osteogenesis. In the postoperative period (3, 5 and 7 days), moderate edema and hyperemia were noted with their complete leveling by the 7th day (*p* < 0.05). According to the histological examination, 3 months after the installation of the titanium mesh, the formation of dense connective tissue with signs of active osteogenesis was observed in the defect area, including zones of mineralized bone trabeculae, osteocytes and osteon elements. The findings of this study indicate acceptable biocompatibility of the developed titanium structure and suggest osteoconductive potential, which, however, needs to be confirmed in controlled, quantitatively powered studies.

## 1. Introduction

Prolonged absence of teeth inevitably leads to atrophy and deformation of the alveolar process of the jaw, which creates serious anatomical and functional limitations for subsequent dental implantation and prosthetics [1]. Restoring the lost volume of bone tissue is one of the key tasks of modern maxillofacial surgery and implantology. Guided bone regeneration is recognized as an effective method of solving this problem, where titanium meshes act as barrier membranes forming a protective contour and space for the implanted osteoplastic material [2].

Despite their widespread use, traditional preformed titanium meshes have several disadvantages. They often do not fully correspond to the individual anatomical features of the bone defect, which can lead to an imperfect fit, migration of the structure, and as a result, unsatisfactory results of osteogenesis. In addition, standard constructions can create difficulties in adaptation and fixation in complex clinical cases, especially in areas with severe deformity [3].

The development of additive technologies has opened up new possibilities for creating customized medical implants that fully match the unique geometry of a particular patient’s defect. The use of 3D printing by selective laser sintering of titanium powder makes it possible to create complex devices with specified biomechanical and biological properties [4].

The use of individualized titanium meshes is particularly relevant in patients with impaired mineral metabolism, in whom the processes of natural regeneration of bone tissue may be slowed down or disrupted. For this category of patients, standard bone grafting methods often prove to be insufficiently effective [5].

In this regard, the purpose of this study was the experimental testing of an individual titanium mesh created by 3D modeling methods for targeted bone regeneration in conditions of vitamin D3 deficiency. This work aimed at studying the features of osteogenesis, the formation of bone regeneration and morphological changes in the area of the defect in the presence of impaired vitamin status. It is assumed that the use of such a mesh can not only stabilize the volume of bone regeneration but also create conditions for more effective osteogenesis in patients with impaired mineral metabolism.

The presented approach demonstrates an increase in the effectiveness of eliminating alveolar deformities by ensuring accurate adaptation, stable fixation and creating optimal conditions for bone regeneration.

## 2. Materials and Methods

### 2.1. Titanium Mesh Design

To achieve a technical result, that is, to eliminate defects and deformations of the alveolar bone, which are often found in the area of prolonged absence of teeth, our partners (Skoltech, Vip-Studio LLC (Bolshoy Boulevard, 30s1, Moscow)) and us have developed a device. The device consists of a lamellar curved frame with a profile in the form of a truncated pyramid, made of titanium powder by sintering on a 3D printer. The upper base of the frame plate is solid, and in the two lateral trapezoidal faces, the frame plates are made with an arbitrary arrangement of holes with a diameter of (0.2–0.5) mm. The upper base and side faces of the frame plate are installed with the possibility of forming an internal space between them to fill it with bone-plastic material on the surface of the alveolar ridge. In addition, holes for mounting fixing screws are located in the lower parts of the side faces of the frame plate, and the thickness of the frame plate is not more than 5 mm (patent RU 212720 U1, 3 August 2022. Application No. 2022107054 dated 17 March 2022).

This article describes in detail the design of the device, the algorithm of its polygonal modeling in the 3D-Materialise software v. 18.0.0.1645 package based on cone beam computed tomography (CBCT) data, as well as the technology of manufacturing titanium powder by 3D printing.

### 2.2. Testing In Vivo

For in vivo testing of an individual titanium mesh, an experimental study was conducted on the basis of the Central Vivarium of the Sechenov First Moscow State Medical University. The model was provided on the adult male Chinchilla rabbits (weight 3.0–3.2 kg) (n = 6). A vitamin D3 deficiency condition was created by placing animals in a separate room without natural light and without introducing vitamin D3-fortified foods for 1 month. To control the level of vitamin D3, venous blood was collected from the ear vein of rabbits into special tubes with reagents and then transferred within 1.5 h to the laboratory for further analysis, which was carried out on Architector 2000 equipment (Center for Molecular Diagnostics (CMD) laboratory) [6].

**Figure 1:**
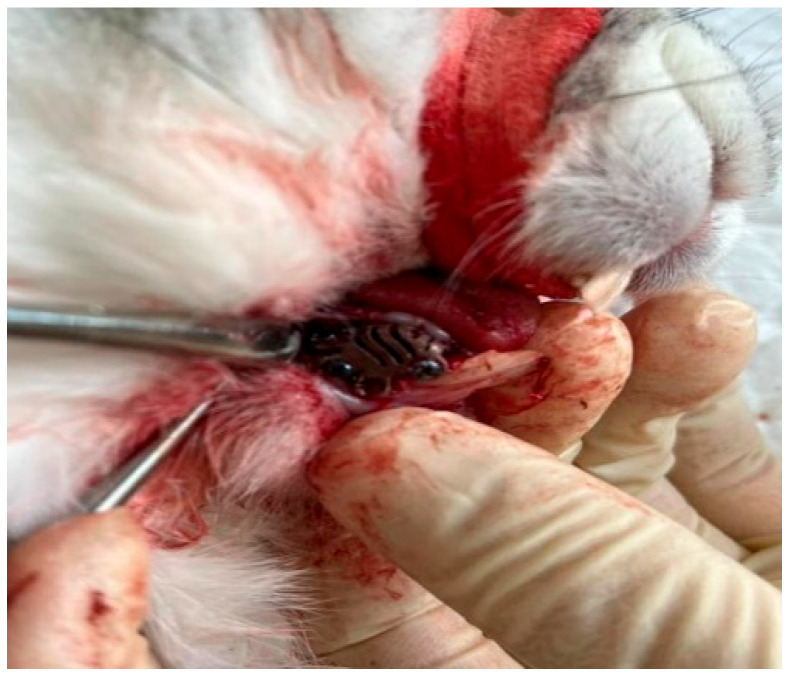
View of an individual titanium mesh in the rabbit’s oral cavity.

Under anesthesia, a bone defect was created in the area of the right half of the lower jaw using a pre-planned 3D model. The formed defect was filled with hydroxyapatite [7], on top of which an individual titanium mesh was installed, fixed with titanium micro-screws (Figure 1). The structure was additionally covered with a collagen membrane [8,9,10], which was followed by the suturing of the tissues without tension. Matriflex Apatite, a mineral matrix of spongy bone tissue of cattle based on hydroxyapatite, completely purified from organic components, crushed and fractionated to particles measuring 0.25–1.00 mm, was used as a bone replacement material. This matrix is characterized by the amorphous (lack of crystallization) hydroxyapatite, as well as the preservation of carbonate ions in the anionic sublattice of the substance, which maximally approximates its chemical properties with the mineral component of native bone. These properties are achieved through chemical technology and thermal purification of bone tissue, excluding heating above 300 °C. For joint use with bone-substituting material, membranes made of highly purified collagen obtained from the dermis of cattle skin were used. Two types of membranes were used: Matriflex Fiber—a thinner membrane with a thickness of 0.3–0.6 mm; and Matriflex Direct, a thicker membrane with a higher density, with a thickness of 0.6–0.8 mm [7,8,9,10]. Follow-up examinations were performed on the 3rd, 5th, 7th and 14th days after surgery. The animals were removed from the experiment after 3 months by overdosing on an anesthetic.

### 2.3. Histological Analysis

Histological analysis was performed in the Laboratory of Digital Microscopic Analysis (Moscow, Trubetskaya str., 8, p. 2, SIC, head–PhD, Associate Professor Fayzullin A.L.). Tissue samples were fixed in 10% neutral buffered formalin, then decalcified in an electrolyte solution based on hydrochloric and formic acids (Ergoprodaktion LLC, Russia) for 14 days. Next, the materials were poured into paraffin blocks with a rigid orientation, providing vertical sections perpendicular to the gum surface. Section 3 and Section 4 microns thick were stained with hematoxylin and eosin. Microscopic analysis was performed using a LEICA DM4000 B optical microscope with a LEICA DFC7000 T digital camera and LAS V4.8 software (Leica Microsystems, Germany). The following factors were assessed: cellular composition, the presence of bone trabeculae in the area of the defect, remnants of bone-plastic material, signs of inflammation (exudation, microbial colonies, neutrophil infiltration, giant multinucleated cells of foreign bodies, etc.), as well as the state of microcirculation.

### 2.4. Statistical Analysis

Statistical data processing was performed using the SPSS Statistics v.26 program (IBM Corp., USA). The nonparametric Friedman criterion was used to evaluate the dynamics of ranked clinical signs (edema and hyperemia) in repeated measurements (3, 5, and 7 days). When statistically significant differences were identified, a pairwise comparison of time points was performed using the post hoc Dunn test with Bonferroni correction. The threshold level of statistical significance (*p*) was assumed to be 0.05. The data in the table is presented as the median (Me), minimum–maximum (Min–Max), and mean ± standard error of the mean (M ± m) to describe the central trend. Given the pilot nature and small sample size of this study (n = 6), all statistical analyses are exploratory and descriptive, and the results should not be interpreted as confirmatory evidence of efficacy.

## 3. Results

### 3.1. Titanium Mesh Design

The choice of the device configuration in the form of a plate-shaped curved frame with a profile in the form of a truncated pyramid with an upper solid base of the frame plate and an arrangement in two lateral trapezoidal faces of the frame plate with an arbitrary arrangement of holes leads to an increase in the efficiency of eliminating alveolar deformation. The implementation of the frame plate from titanium powder by sintering using a 3D printer makes it possible to increase the reliability of the proposed design. The size range of the hole diameters (0.2–0.5) mm, made on the two lateral trapezoidal faces of the frame plate, was established during the research conducted by the authors, and allowed for an increase in the efficiency of eliminating alveolar deformation. The thickness of the frame plate was chosen to be no more than 5 mm because of research, which allowed for an increase in the convenience of surgical intervention.

**Figure 2:**
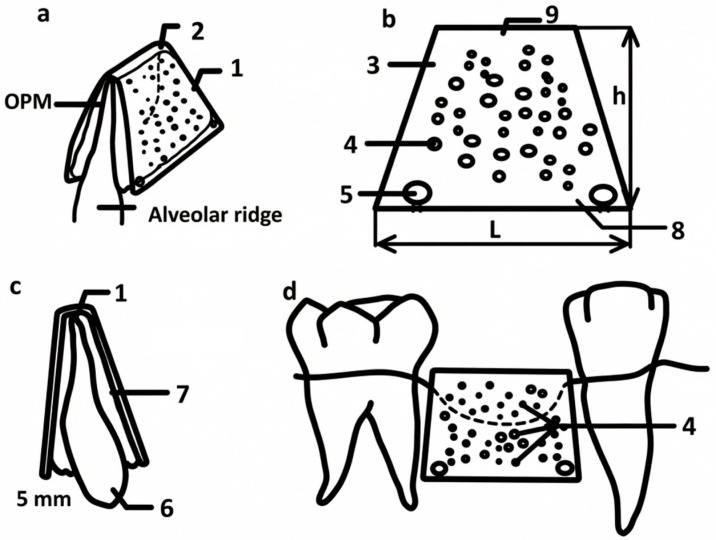
Drawing of a model of an individual titanium mesh in various projections: (**a**) an isometric view of the relative position of the device, the bone-plastic material and the alveolar ridge; (**b**) a view of the lateral trapezoidal face of the frame plate, made with an arbitrary arrangement of holes; (**c**) an additional view of the relative position of the device, the bone-plastic material and the alveolar ridge; (**d**) a view of the relative position of the device and teeth in any area of the jaw. 1—Lamellar curved frame, 2—upper base of the frame plate, 3—lateral trapezoidal face of the frame plate, 4—holes (diameter from 0.2 mm to 0.5 mm) on the side faces of the plate, 5—holes for mounting fixing screws , 6—alveolar ridge, 7—osteoplastic material (OPM), 8—lower base of the lateral trapezoidal face of the frame plate, 9—upper base of the lateral trapezoidal face of the frame plate, h—height of the defect, and L—width of the intervention zone.

The drawing of the model is shown in Figure 2.

**Figure 3:**
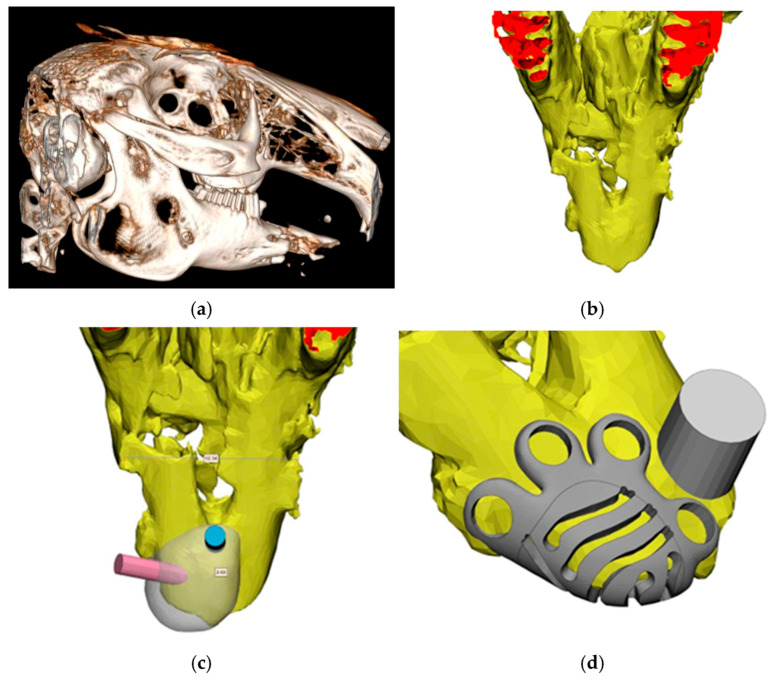

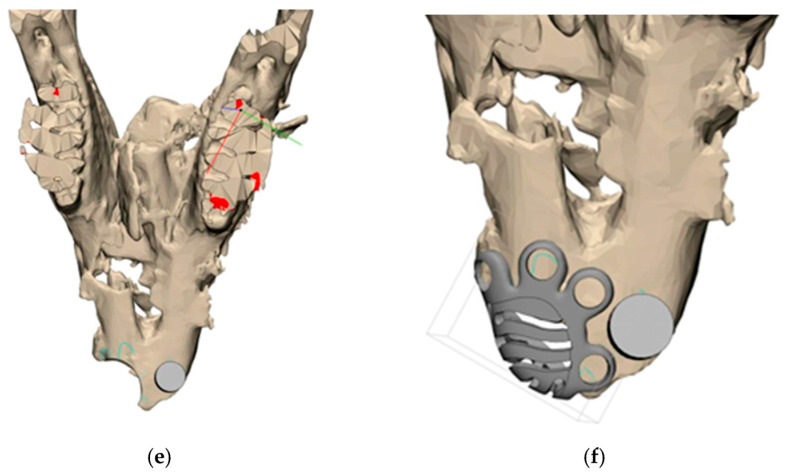
Creation of a virtual model of the defect and an individual titanium mesh to eliminate the defect based on the results of CBCT of the rabbit mandible: (**a**) the CT scan of the rabbit’s head; (**b**) the view of the rabbit’s lower jaw in the 3D-Materialise planning program; (**c**) highlighting the area of interest on the lower jaw; (**d**) creating and modeling an individual titanium mesh in the area of interest on the lower jaw, taking into account the future defect; (**e**) modeling the defect on the lower jaw; and (**f**) checking the compliance of the designed individual titanium mesh defect on the rabbit’s lower jaw.

Prior to the direct printing of the device, an individual device is designed in the 3D-Materialize computer planning system using polygonal modeling based on the results of previously performed cone beam tomography of the patient’s jaws with segmentation of the area of interest. It is possible to maintain the quality of the final result obtained as a result of planning at a high level by registering the dentition and measuring the thickness of the mucosa, which are also determined at the stage of conducting and analyzing the results of cone beam tomography. The tomography scan mode is performed in the “light” mode of the X-ray tube. This technique is presented using the example of a rabbit’s mandible (Figure 3).

**Figure 4:**
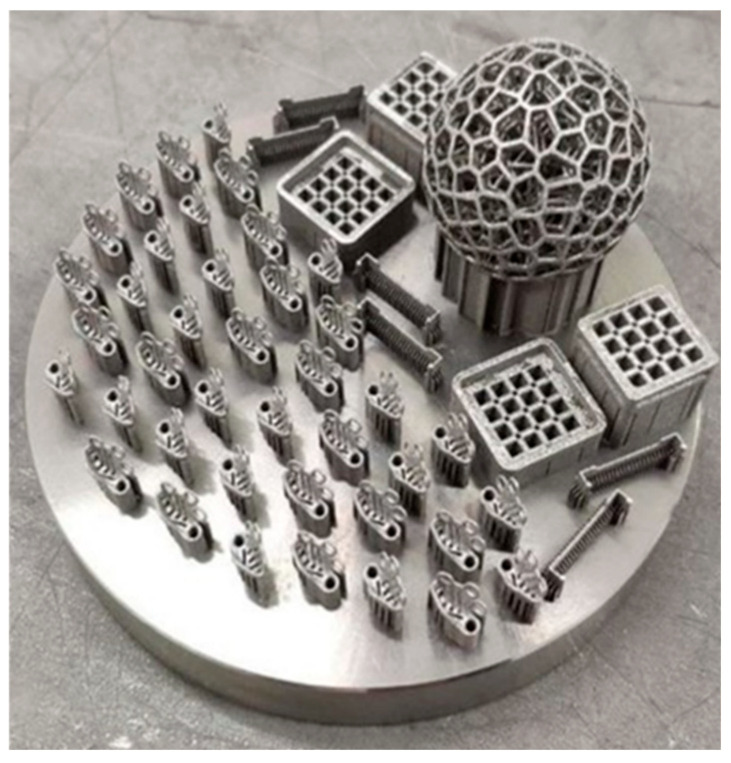
View of an individual titanium mesh after it is printed on a 3D printer. Printed individual meshes are located in the left inner part of the platform.

For the manufacture of the device, a 3D printer is used to print metal products, including titanium powder of one of the brands used in implantology (VT-0, VT-1-0, VT-6, VT-16, etc.). By sintering powder (medium or large with a particle size of up to 150 microns) within 1–2 h, a device of a given shape and size with an installation thickness of no more than 0.5 cm with a perforated structure is obtained—4 holes with 2–3 holes of 0.5 mm diameter for fixing screws to the bone surface (Figure 4).

The dimensions of the device are selected as follows: the size of the lower base 8 consists of the initial width of the alveolar ridge 6 in the area of fixation of the device (plus 2–3) mm on each side of the ridge 6 to accommodate the osteoplastic material 7, and the size of the upper base 9 is the initial width of the alveolar ridge 6 in the area of its tip (facing the oral cavity) (plus 2–3) mm on each side of the ridge 6 to accommodate the osteoplastic material 7. The thickness in all sections is the thickness of the frame plate—no more than 5 mm. The width of the device in the profile is the width of the defect plus 1 mm on each side, but not less than 1–2 mm from the projection of the roots of adjacent teeth. After modeling, printing and making holes 4,5 in the device and after preliminary surgical access with peeling of the mucous-periosteal flaps on both sides of the alveolar ridge 6, decortication is performed (creating perforations in the cortical plate of the alveolar ridge) in the numbers 6 to 10, followed by the placement of osteoplastic material 7 in the area of atrophy and deformation; then, a device is placed on top for elimination of alveolar deformation in the area of tooth loss, which is fixed in the area of the corresponding holes with 5 titanium mini-screws( (Figure 2). Then, the mucous-periosteal flaps are placed on top without tension, and the wound is sutured. The duration of sutures in the oral cavity is 14 days.

### 3.2. Testing In Vivo

During control examinations on days 3, 5, and 7, the development of moderate edema and hyperemia was noted, with their complete leveling by day 7. The results are presented in Table 1.

**Figure 5:**
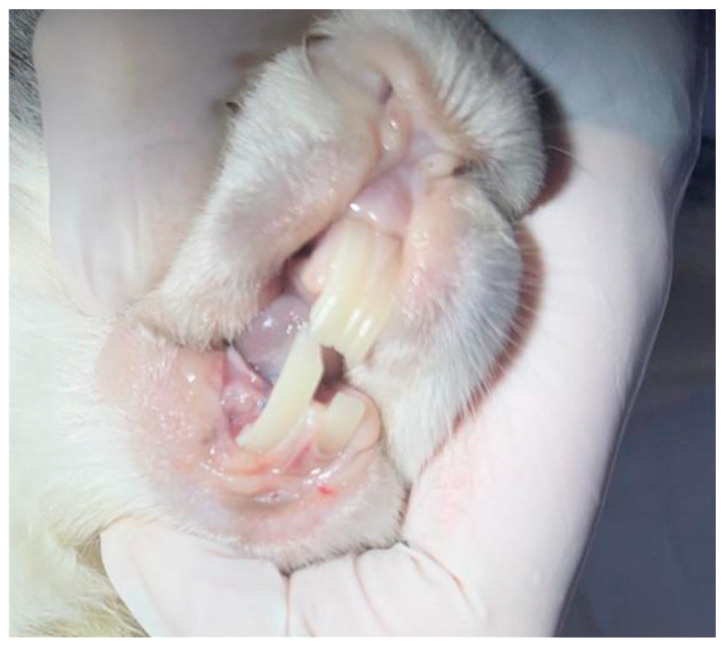
View of the rabbit’s oral cavity three months after inserting titanium mesh.

After three months, the oral mucosa in animals was of normal color without any signs of inflammation or defects (Figure 5).

### 3.3. Histological Analysis

**Figure 6:**
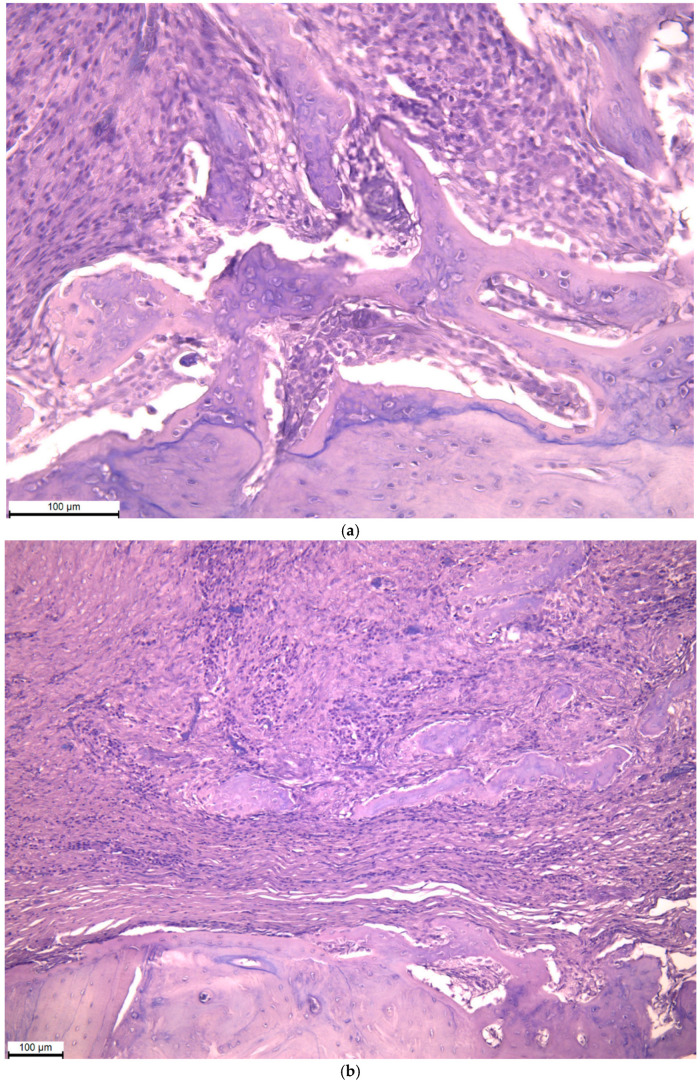
The osteoregenerate area of the lower jaw after removal of the titanium mesh. Hematoxylin–eosin staining: (**a**) ×50; (**b**) ×200.

The micrographs show the area of the defect near the surface of the bone tissue. The defect site is filled with dense connective tissue with a high density of fibroblasts, as well as infiltrated by macrophages and lymphocytes; osteogenesis zones are defined in the form of mineralized beams with osteocytes and signs of osteon formation (Figure 6).

## 4. Discussion

The technique developed in this study represents a specific digital workflow for designing and manufacturing an individualized titanium mesh for guided bone regeneration, which was technically feasible and implementable in a vitamin D3-deficient rabbit model. At this stage, the approach should be regarded as a proof-of-concept rather than a validated solution for complex cases.

The key difference between our approach and the previous one is the transition from unification to personalization. Traditional preformed meshes, being a standard product, often require intraoperative fitting, which violates their integrity and biomechanical properties. Our design, originally designed based on CBCT data, ensures a perfect passive fit to the bone bed. This minimizes micro-mobility, which is one of the main factors provoking bone resorption under the mesh and its subsequent exposure [11]. The geometry in the form of a truncated pyramid is not accidental: it is engineering-based to create and maintain a stable volume under the barrier membrane. The solid upper base reliably protects the osteoplastic material from chewing pressure and the germination of soft tissues, while the perforated side faces guarantee the necessary vascularization and bioactivity [12].

Our method is particularly important when working with patients suffering from disorders of mineral metabolism. In this category of patients, reparative processes are slow, and the risks of bone grafting failure are significantly higher [13]. Standard protocols may not provide sufficient stability and control over the regeneration zone in conditions of potential osteogenesis imperfecta. In this context, our individualized grid acts not just as a barrier but as a highly precise framework that creates perfectly controlled and reproducible conditions for regeneration. It mechanically guides and supports the osteogenesis process, compensating for the patient’s internal metabolic disorders. This makes it possible to achieve a given volume of bone tissue with high predictability according to the literature, which is critically important for subsequent dental implantation without the need for additional, more traumatic operations, such as the transplantation of autogenous bone blocks [14].

The use of selective laser sintering for work with biocompatible titanium alloys ensures not only anatomical accuracy but also reproducible physical and mechanical properties of the product. The optimized diameter of the 0.2–0.5 mm holes, arranged arbitrarily, represents a compromise found during research: it is large enough for effective vascularization and nutrient exchange, but small enough to serve as a barrier to fibroblasts, preventing the prolapse of connective tissue into the regeneration zone [15].

The proposed surgical protocol, including the stage of decortication, is the logical conclusion of the entire technique. Multiple perforations of the cortical plate (6–10) ensure the release of osteoinductive cells and growth factors from the bone marrow spaces, which, in combination with a perfectly fitting skeleton, create a powerful incentive for accelerated ossification [16].

While the use of digital workflow and additive manufacturing to create customized titanium meshes is generally described in the literature [2,4,12], the present study focuses on solving three specific undisclosed tasks. First, a new mesh geometry in the form of a truncated pyramid with a solid upper base and strategically perforated side faces is presented and experimentally substantiated, aimed at optimizing volume stability and vascularization. Secondly, for the first time, the effectiveness of such a personalized design is investigated in conditions of compromised regenerative potential modeled by vitamin D3 deficiency. Thirdly, a complete surgical protocol is proposed and tested, from digital planning and manufacturing to fixation techniques and collagen membrane shielding—within a single methodology.

Although the results of modeling and prototyping are extremely encouraging, long-term randomized controlled trials involving humans, including a special cohort of patients with diagnosed disorders of mineral metabolism, are required to definitively confirm clinical efficacy.

The conducted experimental study made it possible to evaluate the clinical and morphological effectiveness of using an individual titanium mesh for targeted bone regeneration in a model of a bone defect in the lower jaw of a laboratory animal.

Analysis of the data from control examinations in the early postoperative period (days 3, 5 and 7) showed that the installation of the structure is accompanied by a moderate local inflammatory reaction, manifested in the form of edema and hyperemia of soft tissues in the area of intervention. At the same time, these changes were reversible: by day 7, there was a complete leveling of clinical signs of inflammation, which indicates satisfactory biocompatibility of the structure and the absence of signs of infectious complications. A decrease in the severity of edema and hyperemia over time, as reflected by the exploratory statistical analysis, is consistent with an uncomplicated postoperative course and acceptable local tolerance of the device but does not provide confirmatory evidence of safety or efficacy.

The results of histological examination of tissues in the area of the defect 3 months after the installation of the titanium mesh demonstrate a qualitative tissue response at the defect site, with dense connective tissue containing fibroblasts and areas of mineralized trabeculae with osteocytes and early osteon formation. These features are compatible with ongoing osteogenesis but do not allow quantitative conclusions about the extent of bone regeneration or functional integration. Simultaneously detected macrophages and lymphocytes indicate an ongoing but controlled inflammatory reaction typical of tissue restructuring processes under conditions of directed regeneration.

The limitations of the present study should be noted. The main one is the small size of the experimental sample (n = 6), which is typical for pilot studies and is due to the ethical principles of minimizing the use of animals. Although we observed statistically significant positive dynamics of clinical parameters and clear histological signs of regeneration, the quantitative results obtained require confirmation in studies with higher power. This work serves as an important first step, demonstrating the fundamental feasibility and biocompatibility of the approach. Planned further studies will include increased cohorts of animals, control groups (for example, with or without standard grids), and longer follow-up periods to obtain statistically more reliable data. Another limitation is the lack of quantification of the volume and density of newly formed bone using CBCT or histomorphometric analysis. The present study was qualitative and evaluative in the framework of the pilot model. To objectively verify the effectiveness of the method in conditions of vitamin D3 deficiency, further studies are needed with the inclusion of control groups (for example, using standard grids or not having a barrier membrane) and mandatory quantitative analysis, including CBCT to assess the volume parameters of regeneration and histomorphometry to determine the proportion of newly formed bone, residual graft and connective tissue.

Further research may focus on the combination of personalized titanium scaffolds with new generations of bioactive osteoplastic materials modified with nanoparticles, similar to work in related fields [17].

Thus, it can be concluded that the use of an individually designed titanium mesh made by 3D printing makes it possible to effectively form and stabilize the bone grafting zone, contributing to the preservation of the volume of osteoplastic material and the directed growth of new bone tissue. The complexity of clinical and morphological data demonstrates the safety and biological compatibility of the developed design, as well as its potential for wide application in reconstructive maxillofacial surgery.

## 5. Conclusions

This study demonstrated that a personalized titanium mesh with a truncated pyramid-shaped lamellar frame and optimized perforation pattern ensures precise defect contouring, stable fixation and predictable space maintenance in a GBR model under vitamin D3 deficiency. The chosen geometry and size of the mesh openings facilitated adequate vascular ingrowth, reduced soft-tissue compression and supported favorable conditions for osteogenesis within the augmented zone.

In the early postoperative period, only moderate, transient edema and hyperemia were observed, which completely resolved by day 7, indicating good tissue tolerance and high biocompatibility of the developed structure. Histological evaluation at 3 months revealed tissue characteristics consistent with bone formation within the defect area, suggesting osteoconductive potential of the mesh–graft complex under vitamin D3 deficiency; however, these qualitative findings require confirmation in larger, controlled studies with quantitative endpoints.

These findings illustrate three potential advantages of the proposed design that warrant further investigation—namely, improved anatomical fit and mechanical stability, the creation of a microenvironment compatible with guided bone regeneration, and preservation of osteogenesis potential under vitamin D3 deficiency. These potential advantages have not yet been demonstrated in direct comparative studies. The obtained data suggest that such individualized titanium meshes can expand the indications and predictability of GBR in patients with impaired mineral metabolism and may be further translated into clinical practice after additional in vivo and clinical validation.

## 6. Patents

Patent RU 212720 U1, 3 August 2022. Application No. 2022107054 dated 17 March 2022.

## Figures and Tables

**Table 1:** Dynamics of local edema and hyperemia in laboratory animals in the experiment. The statistical significance of the dynamics was assessed using the Friedman criterion followed by post hoc testing (see Section 2.4). Due to the small sample size, *p*-values are reported for descriptive purposes only and should be interpreted with caution.

Criteria	Day 3	Day 5	Day 7	*p*
Edema				
Me ± m	1.67 ± 0.2	1	0	<0.05 (0.00001)
Median	2	1	0	
Min-max	1–2	1	0	
Hyperemia				
Me ± m	1.33 ± 0.52	1	0	<0.05 (0.000042)
Median	1	1	0	
Min-max	1–2	1	0	

## Data Availability

The original contributions presented in this study are included in the article. Further inquiries can be directed to the corresponding author.

## Abbreviations

The following abbreviations are used in this manuscript:

CBCT	Cone beam computed tomography
